# Implementation of exercise countermeasures during spaceflight and microgravity analogue studies: Developing countermeasure protocols for bedrest in older adults (BROA)

**DOI:** 10.3389/fphys.2022.928313

**Published:** 2022-08-09

**Authors:** Eric T. Hedge, Courtney A. Patterson, Carmelo J. Mastrandrea, Vita Sonjak, Guy Hajj-Boutros, Andréa Faust, José A. Morais, Richard L. Hughson

**Affiliations:** ^1^ Schlegel-University of Waterloo Research Institute for Aging, Waterloo, ON, Canada; ^2^ Department of Kinesiology and Health Sciences, University of Waterloo, Waterloo, ON, Canada; ^3^ Research Institute of McGill University Health Centre, McGill University, Montréal, QC, Canada; ^4^ Division of Geriatric Medicine, McGill University Health Centre, McGill University, Montréal, QC, Canada

**Keywords:** spaceflight, bedrest, cardiovascular, musculoskeletal, exercise countermeasures, aging

## Abstract

Significant progress has been made in the development of countermeasures to attenuate the negative consequences of prolonged exposure to microgravity on astronauts’ bodies. Deconditioning of several organ systems during flight includes losses to cardiorespiratory fitness, muscle mass, bone density and strength. Similar deconditioning also occurs during prolonged bedrest; any protracted time immobile or inactive, especially for unwell older adults (e.g., confined to hospital beds), can lead to similar detrimental health consequences. Due to limitations in physiological research in space, the six-degree head-down tilt bedrest protocol was developed as ground-based analogue to spaceflight. A variety of exercise countermeasures have been tested as interventions to limit detrimental changes and physiological deconditioning of the musculoskeletal and cardiovascular systems. The Canadian Institutes of Health Research and the Canadian Space Agency recently provided funding for research focused on Understanding the Health Impact of Inactivity to study the efficacy of exercise countermeasures in a 14-day randomized clinical trial of six-degree head-down tilt bedrest study in older adults aged 55–65 years old (BROA). Here we will describe the development of a multi-modality countermeasure protocol for the BROA campaign that includes upper- and lower-body resistance exercise and head-down tilt cycle ergometry (high-intensity interval and continuous aerobic exercise training). We provide reasoning for the choice of these modalities following review of the latest available information on exercise as a countermeasure for inactivity and spaceflight-related deconditioning. In summary, this paper sets out to review up-to-date exercise countermeasure research from spaceflight and head-down bedrest studies, whilst providing support for the proposed research countermeasure protocols developed for the bedrest study in older adults.

## Introduction

Spaceflight causes marked changes to the musculoskeletal and cardiovascular systems like those observed during prolonged inactivity. Accordingly, even early human spaceflight incorporated exercise countermeasures aimed at preventing physical deconditioning (Hackney et al., 2015). Exercise equipment during early short-duration flights (Gemini and Apollo missions) was small and simple in design (e.g., bungee exerciser) (Hackney et al., 2015). Aerobic exercise equipment was available during Space Shuttle flights, and exercise was recommended without prescribed intensity or duration for every second or third mission day (Scott et al., 2019); however, some shuttle astronauts did not exercise while in orbit (Edgerton et al., 1995). More advanced equipment with greater loading capabilities was utilized during longer-duration missions, and more recently aboard the International Space Station (Hackney et al., 2015). Current International Space Station (ISS) exercise hardware includes a cycle ergometer (CVIS), treadmill (T2), and advanced resistive exercise device (ARED) (Korth, 2015). Astronauts on-board the ISS are typically allocated 2.5 h of total exercise time per day, which consists of 1.5 h of resistance training and 1 h aerobic training (Loehr et al., 2015). Importantly, the actual time spent performing exercise each day is much less (Fraser et al., 2012), as the allotted time also includes equipment set-up and stowage, and personal hygiene (Loehr et al., 2015).

Six-degree head-down bedrest (HDBR) is widely used as an experimental model to simulate the effects of microgravity and evaluate the effectiveness of different exercise countermeasures before implementing them during spaceflight; however, there are some notable differences between spaceflight and HDBR related to fluid shifts (see Table 1), spinal dysfunction and radiation exposure (Hargens and Vico, 2016). Different exercise countermeasures have been tested during HDBR, such as aerobic (Stremel et al., 1976; Greenleaf et al., 1989) or resistance training protocols (van Duijnhoven et al., 2010b), novel exercise modalities (Kramer et al., 2017a), and combinations of exercise with fluid loading (Shibata et al., 2010), artificial gravity (Watenpaugh et al., 2000; Iwasaki et al., 2005; Schneider et al., 2009) or whole-body vibration (van Duijnhoven et al., 2010b; Coupé et al., 2011; Guinet et al., 2020). In addition to aerospace medicine research, bedrest experimental models are also useful for examination of the rapid physiological deconditioning that occurs during prolonged hospitalization and inactivity on Earth.

**TABLE 1:** Comparison of the effects of spaceflight and head-down bedrest (HDBR) on blood volume (+: increased, −: decreased). Adapted and modified from Diedrich et al. (2007).

	Spaceflight	HDBR
Headward fluid shift	+	+
Hunger	−	+/-
Thirst	−	+/-
Diuresis	+/-, (explained above)	+
Renal response to fluid & salt	Reset	Preserved
Salt retention	+	Unknown
Atrial natriuretic peptide	Reset to lower levels	+
Mechanical compression of thorax	−	Maintained
Central venous pressure	−	Transiently increased
Radiation	+	NA

An important disconnect exists between those who typically participate in HDBR studies and astronauts. The median age of bedrest study participants is 24.5 years (inter-quartile range: 22.4–34.0 years) (Ried-Larsen et al., 2017), whereas the mean age of astronauts for first and last flights are 40.9 years (maximum: 58.8 years) and 45.3 years (maximum: 61.3 years, excluding John Glenn at 77.3 years), respectively (Kovacs and Shadden, 2017). Both spaceflight and HDBR studies are infrequent, with very limited investigations of older adults. Accordingly, an important knowledge gap exists on the effects of HDBR, and exercise countermeasures on middle-aged and older adults.

**FIGURE 1:**
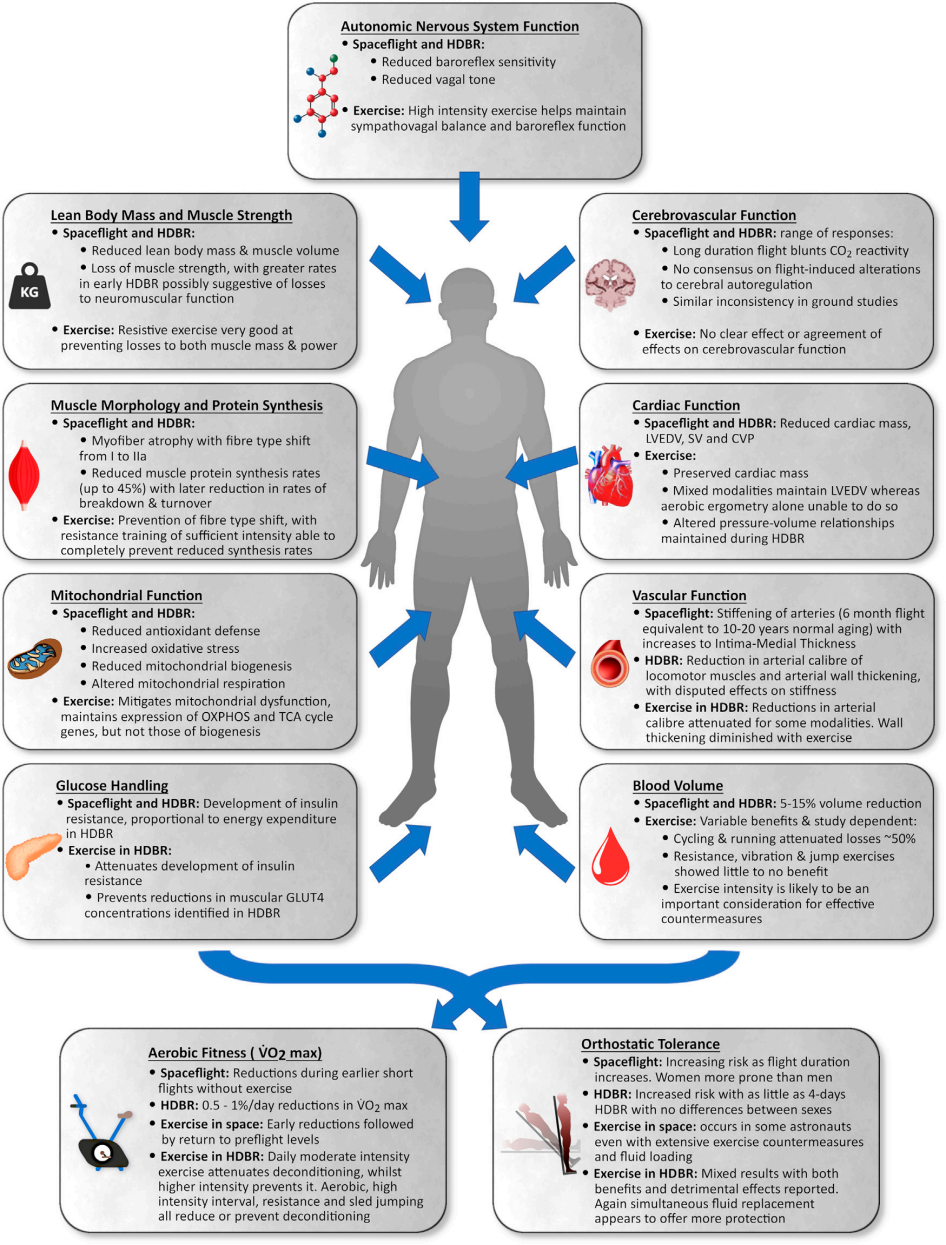
Summary of the impact of spaceflight and head-down tilt bedrest (HDBR) in young to middle-aged adults and the impact of exercise as a countermeasure on various portions of the cardiovascular and musculoskeletal system.

The purpose of this review is to provide an overview of the musculoskeletal and cardiovascular responses to spaceflight, bedrest, and aging, as well as how exercise can mitigate deconditioning in these systems. There is a lack of knowledge regarding HDBR-induced deconditioning in older adults, and we draw attention to knowledge-gaps for physiological systems explored in this review. Furthermore, we introduce the BROA study and discuss the rationale and development of a multimodal exercise countermeasure protocol that will be utilized during this two-week HDBR in older adults aged 55–65 years. Figure 1 provides an overview of the knowledge reviewed in this paper.

## Exercise countermeasures employed during the WISE-2005 study

We will refer to this study throughout this review. The WISE-2005 study was a 60-day HDBR that combined supine squats (4 sets of 7 maximal repetitions) and calf presses (4 sets of 14 maximal repetitions) 2–3 times/week, with aerobic vertical treadmill exercise in lower-body negative pressure (LBNP) calibrated to 1.0x body weight 2–4 days/week with steps up to 80% peak oxygen uptake ( $\dot{V}$ O_2_).

## Lean body mass and muscle strength

### Spaceflight

**FIGURE 2:**
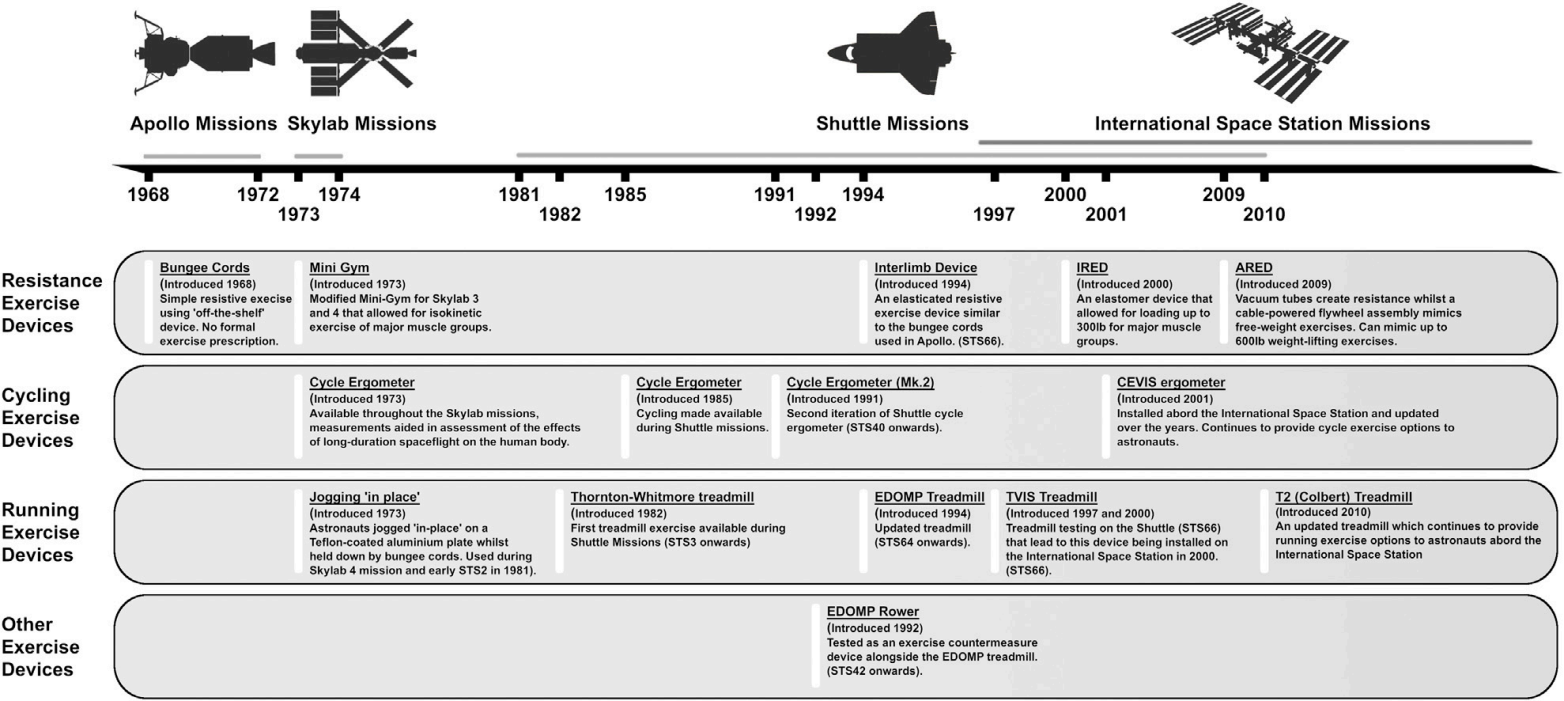
Timeline overview of exercise equipment available on Apollo, Skylab, Shuttle and ISS missions from 1968 to present day.

Gravitational unloading during spaceflight results in loss of lean body mass and muscle strength. During early Skylab missions, lower-limb muscle volume estimated by leg circumference (↓∼5%) and muscle strength (↓∼25%) were reduced (Thornton and Rummel, 1977). Subsequently, studies confirmed muscle losses following short-duration flights in the major muscles of the lower limbs (LeBlanc et al., 1995; Akima et al., 2000) and trunk (LeBlanc et al., 1995). These losses led to reductions in trunk (↓23%) and lower-body strength (↓12%) (Greenisen et al., 1999). The introduction of regular exercises, improvements in exercise equipment and introduction of new exercise modalities (Figure 2), as well as increases to exercise duration (∼30 W-mins/day during Skylab 2 to ∼70 W-min/day during Skylab 4) attenuated but did not prevent muscle losses during Skylab missions (Thornton and Rummel, 1977). Even during early missions to the ISS, losses to lower-limb muscle mass and strength persisted, despite regular exercise countermeasures (Trappe et al., 2009; Gopalakrishnan et al., 2010). It was not until the introduction of the T2 and ARED devices that astronauts were able to maintain proportional lean body mass during their flights (Smith et al., 2012). Most recently, NASA’s SPRINT study concluded that a combination of resistance training (3 days/week) and a mix of high-intensity interval and continuous aerobic training (6 days/week) was just as effective as standard countermeasure regimes at preventing loss of lean leg mass and strength, requiring 33% less time to complete (English et al., 2020). A recent systematic review determined that current countermeasure exercises, particularly resistance exercises using newer equipment, are crucial in preventing muscle losses during spaceflight, although complete prevention is still not possible (Moosavi et al., 2021).

### Bedrest studies

Muscle size and strength losses during bedrest studies are similar to those induced by spaceflight (Narici and de Boer, 2011), with lower-limb and trunk musculature generally affected more than upper-limb muscles (Winnard et al., 2019). Both muscle size and strength followed a nonlinear logarithmic decay during bedrest, with higher rates of change initially that slow over time (Marusic et al., 2021). The ratio of proportional strength loss to muscle atrophy was elevated initially during bedrest before plateauing by ∼35 days, suggesting factors other than muscle atrophy largely contribute to early reductions in strength; however, the vast majority of reductions to muscle strength during bedrest can be explained by muscle atrophy (Marusic et al., 2021).

During a 17-week horizontal bedrest, an exercise countermeasure consisting of progressive upper- (3 days/week) and lower-body (3 days/week) resistance exercises starting at 2 sets and increasing to 6 sets by the end of the campaign, with each set consisting of 11 repetitions at 74% of a 1-repeition maximum, and heel raises (6 days/week), with the same set and repetition scheme as the other exercises on lower-body days and a 5-set, 20 repetition protocol at 58% of 1-repeition maximum on upper-body days, attenuated but did not prevent muscle volume losses, despite improving isotonic strength (Shackelford et al., 2004). Muscle atrophy and strength losses during HDBR were also attenuated by resistive vibration exercise (Rittweger et al., 2010), or aerobic treadmill exercise with body weight loading (Cavanagh et al., 2016). In the WISE-2005 study, again muscle strength increased whilst volume losses were attenuated in exercisers during 60 days of HDBR (Trappe T. A. et al., 2007). A comparison of the type of exercise device (traditional resistance vs. flywheel) found little difference in muscle outcomes between groups (Ploutz-Snyder et al., 2018). Only one set of exercise countermeasures at NASA’s Flight Analogues Research Unit (FARU) during HDBR prevented volume losses completely in targeted muscles (3 days/week of resistance exercises comprising 3 sets of 12 reps at 10-repetition maximums loading for supine squat, leg press, heel raise, and leg curl, and alternating days supine cycling switching between continual aerobic at 80% of peak $\dot{V}$ O_2_ or interval training up to maximal effort) when delivered over a 14-day HDBR in which muscle strength also increased (Ploutz-Snyder et al., 2014). Improvements in participants’ technique and neural adaptation to resistance training may account for the improvements in strength without increases to muscle volume. However, the clear benefit of exercise on both volume and strength compared to control participants clearly supports efficacy of exercise training during HDBR and periods of immobility. Furthermore, the frequency of the intervention is important; over 90 days of HDBR, supine squats (4 sets of 7 maximal repetitions) and calf presses (4 sets of 14 maximal repetitions) every third day (starting HDBR day 5) only attenuated reductions to lower-limb muscle cross-sectional area (CSA; ↓25.6% in control vs. ↓17.3% in exercise) (Rittweger et al., 2005), which is a substantially smaller benefit compared to the aforementioned studies. In summary, exercise, particularly regular resistance exercise, can attenuate muscle volume losses and even improve strength during both supine and HDBR.

### Older adults

Increasing age may accelerate the rate of bedrest-induced muscle loss (English and Paddon-Jones, 2010). Reductions in lean body mass and strength were found following as little as 10 days of sedentary bedrest in older adults (Kortebein et al., 2008; Coker et al., 2015). Comparison of 14-day supine bedrest in older (55–65 years) vs. younger (18–30 years) adults identified more detrimental effects on muscle power in the older age group, identifying important differences in their responses to disuse (Rejc et al., 2018). We identified one seven-day bedrest study in older adults with a 2000 steps/day exercise intervention, but the countermeasure did not prevent loss of muscle mass or strength (Arentson-Lantz et al., 2019). Therefore, formal evaluation of exercise countermeasures in older adults is paramount, especially given that loss of muscle mass and strength results in a significant reduction in functional ability (Kortebein et al., 2008; Coker et al., 2015), potentially leading to decreased ability to perform activities of daily living and a loss of independence.

## Muscle morphology

### Spaceflight

Exposure to microgravity reduces skeletal muscle size, function, and metabolic capacity by inducing changes at the myocellular level. Spaceflight-induced fiber type shifts from type I (oxidative) to type IIa (oxidative/glycolytic) are known to occur during both short- (Edgerton et al., 1995; Zhou et al., 1995; Widrick et al., 1999) and long-duration flights (Trappe et al., 2009; Fitts et al., 2010), with concomitant increases in co-expressing fibers (Widrick et al., 1999, Widrick et al., 2001; Trappe et al., 2009). Combined with reductions in myofiber CSA, as noted in the vastus lateralis (VL) (Edgerton et al., 1995) and soleus muscles (Widrick et al., 1999, Widrick et al., 2001; Trappe et al., 2001), spaceflight causes serious detrimental effects to muscle function. This can lead to greater susceptibility to muscle fatigue, decrease of muscle strength and speed of muscle contraction in astronauts potentially limiting their work capacity during spaceflight. Despite very limited available data in astronauts due to concerns regarding complications from repeated muscle biopsies, we do know that these changes occur even when aerobic exercise or resistance exercise devices, such as iRED (See Figure 2), are available (Trappe et al., 2009). Evidence suggests that the magnitude of myofiber losses are inversely related to the intensity of in-flight aerobic exercise (Fitts et al., 2010), but definitive assessment of optimal countermeasure protocols to combat these losses does not yet exist.

### Bedrest studies

As with spaceflight, detrimental morphological muscle changes occur during bedrest. Previous studies identified reductions in CSA of type IIa myofibers in VL muscle following 10-day supine bedrest (Standley et al., 2020), and of all myofiber types in the VL after 35- and 37-day HDBR (Andersen et al., 1999; Borina et al., 2010; Brocca et al., 2012). Following 60-day HDBR, CSA of type I and IIa myofiber types in soleus and VL muscles were reduced (Salanova et al., 2014). Over a 122-day HDBR campaign, Ohira et al. observed progressive reductions to type I myofiber CSA in the soleus, with 12% losses at the halfway point, and 39% losses by the end of the study (Ohira et al., 1999). These reductions in CSA tended to be accompanied by changes in myofiber type (Ohira et al., 1999). Shifts away from type I and towards type IIx co-expressing myofibers was noticed in protein analyses of the VL muscle following 35-day HDBR (Borina et al., 2010; Brocca et al., 2012), whilst Andersen et al. identified corroborating changes in mRNA expression of myosin heavy chain protein genes without difference in protein levels following 37 days of HDBR (Andersen et al., 1999). Decreases in the proportion of type I myofibers in both the soleus and VL muscles, and an increase in the proportion of co-expressing myofibers were noted in the 60-day HDBR campaign (Salanova et al., 2014). Myofiber atrophy commences soon after the initiation of bedrest and can gradually progress for at least 4 months (Ohira et al., 1999), but beyond this time point no data exists. Therefore, similar morphological changes occur between spaceflight and bedrest studies.

Regarding exercise countermeasures, the combination of aerobic exercise with LBNP and resistance training, resistance exercise and vibration, or just resistance exercise during 60 days of HDBR, maintained myofiber CSA and further increased the CSA of type IIa myofibers in both soleus and VL muscles (Trappe S. et al., 2007; Salanova et al., 2008, Salanova et al., 2014). Resistance training 2–3 days/week during an 84-day HDBR campaign (4 sets of 7 maximal supine squats) preserved type IIa myofiber composition and size in VL muscle, and type IIa and type I myofiber composition in soleus muscle (Trappe et al., 2004; Gallagher et al., 2005). However, both the exercise and control groups in this study displayed an increased proportion of co-expressing myofibers in these muscles (Trappe et al., 2004; Gallagher et al., 2005). More recently, Blottner et al. (2020) reported an almost completely protective effect of head-down sled jumping for both type I and II fibers’ CSA in the soleus muscle, with no myofiber shifts in the VL despite a downward trend in the proportion of type I fibers in the soleus. The high load jumping (80%–90% bodyweight) provided over 48 exercise sessions during the 60-day campaign was an effective countermeasure for HDBR-induced muscular changes noted in the VL and soleus of control participants (Blottner et al., 2020).

Therefore, exercise attenuates, and may even prevent, the detrimental effects of HDBR on muscle morphology and serves as an analogue to spaceflight studies. Exercise programs during HDBR have the capacity to counteract some of the detrimental effects at the myocellular level; however, these studies focus on muscles of the lower limb, and countermeasure programs must be more holistic.

### Older adults

Muscle mass and strength generally diminish with age, with concomitant myofiber atrophy (Lexell et al., 1988; Lexell and Taylor, 1991). Myofiber atrophy experienced with aging is exacerbated by physical inactivity (Arentson-Lantz et al., 2016; Pišot et al., 2016) and were not attenuated with a 2000-daily steps intervention (Arentson-Lantz et al., 2019). Given that older adults typically have less muscle, and it declines more quickly with inactivity, dedicated studies are needed to determine optimal exercise countermeasures in this special population to prevent muscle wasting during periods of immobility.

## Muscle protein synthesis and breakdown

### Spaceflight

Muscle protein mass represents the net balance between rates of muscle protein synthesis (MPS) and muscle protein breakdown (MPB), whose turnover rates are constantly in a dynamic state of adjustment, allowing the body to maximize protein balance when facing limited supply of amino acids (Stein, 1999; Morais et al., 2018). On immediately entering microgravity, nausea, vomiting and space motion sickness lead to stress-induced reductions in energy and protein intake, as well as negative nitrogen balance (Stein et al., 1993, Stein et al., 1996; Stein and Schluter, 1994). This is followed by an abrupt increase in MPB by flight day 3, and reductions to both MPS and MPB by flight day 6 (Ferrando et al., 2002), which supports historical measurements of negative nitrogen balance during Skylab missions (Whedon et al., 1977). Following the initial dynamic response to environmental changes, astronauts’ muscle protein turnover appears to be reduced (Stein et al., 1999a; Ferrando et al., 2002). Indeed, there is decreased whole body protein synthesis during long-duration spaceflight (Stein et al., 1999a) and short-duration missions (Ferrando et al., 2002). In the later study, reduced MPB was also observed based on the urinary excretion of 3-methyl-histidine (Ferrando et al., 2002). These results suggest physiological adaptation to a state in which the body cannot maintain MPS at the same rate in skeletal muscle, likely due to reductions in activity and muscle loading, as well as an energy deficit. Therefore, exercise plays an important role in stimulating muscle protein turnover after the first days of spaceflight and exemplifies the need for adequate exercise countermeasures for astronauts. To the best of our knowledge the effect of aerobic and resistance exercise on muscle protein kinetics during spaceflight is yet to be studied.

### Bedrest studies

Bedrest without countermeasures has detrimental effects on muscle protein kinetics. Fourteen-day (Ferrando et al., 1996) and 21-day HDBR (Symons et al., 2009) both reduced MPS without changing MPB. Few investigations have studied MPS and MPB during bedrest with exercise countermeasures. There is some evidence to suggest exercise is protective of muscle protein kinetics, as resistive knee extension exercises (3 sets of 10–12 repetitions/day with progressive daily loading) maintained lower-body MPS during 14-day HDBR (Ferrando et al., 1997). Sedentary bedrest studies consistently show muscle protein changes are induced by lower MPS rates whereas protein breakdown seems to be affected to a lesser degree (Ferrando et al., 1996; Symons et al., 2009). Exercise, especially resistance type mitigates such loss, but it needs to have a progressive overload nature to achieve protective effects on MPS and MPB (Ferrando et al., 1997). Further research in this field focusing on muscle protein metabolism, including changes in kinetics and cellular signalling proteins, and the impact of countermeasure exercises during HDBR are required to fully understand how exercise may provide beneficial results in immobile or weightless individuals.

### Older adults

Aging is associated with anabolic resistance to MPS in the ambulatory state as well as during bedrest, which predisposes older adults to increased muscle atrophy compared with their younger counterparts. Older adults appear to be at a higher risk for muscle loss during bedrest, as two-week supine bedrest increased anabolic resistance to a standardized meal four times more in older (∼59 years) compared to younger adults (∼23 years) (Biolo et al., 2017). Similarly, older adults (∼66 years) had diminished post-prandial fractional protein synthesis rates and elevated markers of muscle proteolysis with reduced lower-limb muscle mass and strength following five days of supine bed rest, while younger adults (∼22 years) were largely unaffected (Tanner et al., 2015). Exercise can combat impaired MPS in older adults (Dickinson et al., 2013), but the efficacy of exercise countermeasures to preserve MPS in older adults during bedrest has not been evaluated.

## Mitochondria

### Spaceflight

Mitochondria are implicated in several important cellular functions, including energy transduction, activation of apoptotic signaling, and regulation of reactive oxygen species (ROS) production (Hepple, 2016). These processes modulate a wide variety of cell signaling pathways, including those associated with muscle atrophy (Romanello et al., 2010). Spaceflight leads to increases in mitochondrial ROS production whilst also altering expression of genes related to adenosine triphosphate generation pathways in human fibroblasts (da Silveira et al., 2020). Within the muscle, no change in succinate dehydrogenase activity, a marker of myofiber oxidative capacity was found in the VL muscle from astronauts after a 11-day spaceflight (Edgerton et al., 1995). After a six-month spaceflight incorporating iRED, CEVIS and treadmill running countermeasures between 2002–2005 (Figure 2), the activity of cytochrome oxidase of complex IV of the electron transport chain was reduced by 59% in type I fibers of both soleus and gastrocnemius muscle of astronauts performing less treadmill training (<100 min s/week) compared to no change in those running for longer (Fitts et al., 2013). There was no change in these enzyme activities in type IIa fibers, nor were there any changes to activity of mitochondrial enzymes *β*-hydroxyacyl-CoA dehydrogenase and citrate synthase (CS), representing *β*-oxidation and tricarboxylic acid (TCA) cycle activity respectively, in soleus or gastrocnemius muscle independent of running duration (Fitts et al., 2013). This may be due to the high heterogeneity in the activity of given enzymes within a particular fiber, muscle type and the low number of subjects studied (Fitts et al., 2013). Nevertheless, the activity of *β*-hydoxyacyl-CoA dehydrogenase and CS was comparatively higher in soleus type I fibers of the high-intensity treadmill group post-flight (Fitts et al., 2013). It seems that appropriate in-flight exercise programs (duration, mode, intensity) can stimulate muscle aerobic enzyme activity (Fitts et al., 2013) which is important for maintenance of normal mitochondrial function. However, the role of in-flight exercise on muscle mitochondrial function requires further investigation.

### Bedrest studies

After 14 days of supine bedrest, protein levels of PGC-1a, a master regulator of mitochondrial biogenesis (Popov, 2020), and Sirt3, a protein involved in mitochondrial activity (Vassilopoulos et al., 2014), were significantly reduced (Buso et al., 2019). Similarly, expression of eight subunits of CI, CII, CIV, and CV was reduced in addition to two subunits of the TIM/TOM complex (Buso et al., 2019), which is important for protein translocation through the mitochondrial membrane (Gomkale et al., 2021). After 10 days of supine bedrest, RNA-sequencing revealed reduction in the expression of genes involved in the TCA cycle and mitochondrial respiration measured in permeabilized myofibers, whilst ROS production increased, likely driven by reductions in mitochondrial content (Standley et al., 2020). Similar findings were observed after 21 days of supine bedrest and HDBR, where significant reduction in adenosine diphosphate-stimulated mitochondrial respiration and maximal aerobic capacity were found in VL muscle of young subjects (Kenny et al., 2017; Salvadego et al., 2018). Following 60 and 84 days of HDBR, gene expression analysis of VL and soleus muscle showed reduced expression of genes involved in TCA cycle, oxidative phosphorylation, lipid metabolism and mitochondrial biogenesis (Salanova et al., 2015; Irimia et al., 2017; Fernandez-Gonzalo et al., 2020).

With regard to exercise countermeasures, participants completing resistance (squats and heel raises) vibration (25 Hz) exercise training on five days during 21-day HDBR did not exhibit alterations in mitochondrial respiration (Kenny et al., 2017). Resistance exercise (squats) 2–3 days/week during 84 days of HDBR was not enough to preserve gene expression of some mitochondrial biogenesis markers (Irimia et al., 2017). However, resistance exercise intervention was able to offset alteration of gene expression related to oxidative phosphorylation, TCA cycle and fatty acid oxidation (Fernandez-Gonzalo et al., 2020). Finally, rehabilitation comprising a multimodal exercise program following 14 days of supine bedrest restored the protein levels of PGC-1a and Sirt3, and CS activity (Buso et al., 2019). However, only protein levels of complexes CII and CIII recovered to baseline values, whereas the expression of CV did not recover after the rehabilitation period (Buso et al., 2019). The exercise program upregulated genes involved in oxidative phosphorylation and TIM/TOM complex (Buso et al., 2019). These findings suggest that exercise during or immediately following bedrest can mitigate some of the negative effects of inactivity on mitochondrial function and other metabolic markers.

### Older adults

Short duration supine bedrest (10 and 14 days) in older subjects reduced protein levels of almost all oxidative phosphorylation complexes (CI, CII, CIII, CIV, and CV) (Buso et al., 2019; Standley et al., 2020). Additionally, it reduced activity of CS, which is often used as marker of mitochondrial content (Buso et al., 2019). These detrimental effects were far greater than those identified in younger adults participating in the same study. Therefore, older populations (55–65 years) respond differently compared to younger populations, with a far greater impact of bedrest on mitochondrial function. However, so far as we are aware, no studies have yet investigated the effects of countermeasure exercises on mitochondrial function in older adults during HDBR.

## Changes in cardiac function

### Spaceflight

During early human space exploration, limited or non-existent availability of exercise modalities resulted in marked changes in cardiac function despite the relatively short exposure to microgravity. Following Apollo, Spacelab, and early Space Shuttle missions supine resting heart rate (HR) was increased in astronauts upon returning to Earth (Hoffler and Johnson, 1975; Bungo et al., 1987; Buckey et al., 1996b) whereas supine cardiac output (Q̇) and stroke volume (SV) were decreased (Hoffler and Johnson, 1975; Bungo et al., 1987; Levine et al., 1996). Heart size (Hoffler and Johnson, 1975), left ventricle end diastolic volume (LVEDV) (Henry et al., 1977; Bungo et al., 1987) and LV mass (Perhonen et al., 2001a) were also reduced post-spaceflight. In microgravity, central venous pressure (CVP) is lower than supine posture, but the enhanced transmural pressure gradient increases cardiac filling (Buckey et al., 1996a; Lawley et al., 2017) resulting in increased SV and cardiac output while in space (Norsk et al., 2015; Hughson et al., 2017).

The extent of cardiac changes with longer duration space missions is influenced by the overall reduction in daily energy expenditure (Fraser et al., 2012) balanced to variable degrees by exercise countermeasures (Moore et al., 2010; Fraser et al., 2012). Immediately following long-duration spaceflight missions on Mir space station, reduced SV, ejection fraction and percent fractional shortening were observed (Martin et al., 2002). Currently, the combination of in-flight aerobic exercise and resistance training appears sufficient to maintain cardiac function assessed *via* heart-rate responses to normal daily living (Fraser et al., 2012). Transient prolongation of left ventricular ejection time post-flight and slightly increased resting HR (Hughson et al., 2012), appear to resolve quickly (Vandeput et al., 2013), consistent with adequate cardiac protection from current ISS countermeasure activities. Furthermore, maintenance of seated SV and Q.(Hughson et al., 2012), and cardiac mass and LVEDV (Abdullah et al., 2018) following 4–6 months of spaceflight support this conclusion. It should be noted that left atrial volume increased in astronauts performing these same exercise countermeasures; however, this resolved quickly post-flight and did not result in any change in atrial function or increase in supraventricular arrhythmogenic potential (Khine et al., 2018). Importantly, with more advanced equipment and adequate exercise duration and intensity, cardiac function following spaceflight appears to be protected.

### Bedrest studies

HDBR induces changes to cardiac function that are like those identified during spaceflight. Elevated resting supine HR following HDBR of 35 days (Hastings et al., 2012) and 60 days (Edgell et al., 2007), and reduced resting supine SV in studies of greater than two weeks (Levine et al., 1997; Perhonen et al., 2001a; Kozàkovà et al., 2011; Hastings et al., 2012), indicate significant cardiac consequences of the HDBR intervention. Similarly, losses to cardiac mass (Levine et al., 1997; Perhonen et al., 2001a; Kozàkovà et al., 2011), reductions to LVEDV (Levine et al., 1997; Perhonen et al., 2001a; Kozàkovà et al., 2011), and decreased LV distensibility contribute to a loss of cardiac function (Levine et al., 1997; Kozàkovà et al., 2011), with LV untwisting also noted to slow (Dorfman et al., 2008). The noted fall in CVP in spaceflight was also observed during HDBR at both the 4 h (Fischer et al., 2007) and two-week time points (Levine et al., 1997).

The intensities and modes of aerobic and resistance countermeasures during HDBR in combination with the subjects’ activity levels prior to bedrest contribute to observations of variable cardiac responses (Dorfman et al., 2007; Shibata et al., 2010; Yang et al., 2011; Hastings et al., 2012; Li et al., 2017; Scott et al., 2018; Greaves et al., 2019). When compared to control participants, countermeasure exercises during the WISE-2005 study maintained HR, SV, and LVEDV and cardiac mass during HDBR (Dorfman et al., 2007; Edgell et al., 2007), as did exercises without concomitant LBNP during a 70-day HDBR study at the NASA FARU facility (see description of exercises in lean body mass and muscle section) (Scott et al., 2018). Rowing exercise (6 sets of 3-min high-intensity intervals) combined with upper- and lower-body resistance exercises also mitigated cardiac deconditioning (Hastings et al., 2012). Other benefits of these concurrent exercise countermeasures included preserved ventricular compliance (Hastings et al., 2012), and cardiac mechanics (LV strain and twist) (Scott et al., 2018). Similarly, resistive vibration exercise prevented changes in LVEDV, LV mass and contractility following 21-day HDBR (Greaves et al., 2019). Supine cycling alone (3 sessions x 30 min/day at 75% maximum HR) attenuated reductions to LVEDV and SV whilst maintaining cardiac mass and diastolic suction over 18 days of HDBR (Dorfman et al., 2008; Shibata et al., 2010), whilst supine cycling at only 40 W and combined with artificial gravity at alternating 1–2 Gz (foot level) preserved resting supine HR and systolic function during 4-day HDBR (Yang et al., 2011; Li et al., 2017). Overall, most HDBR studies provide evidence that aerobic and/or resistance exercise are beneficial for maintaining cardiac morphology and function, but differences in cardiac responses between concurrent and aerobic only countermeasures suggest the type and intensity of exercise is important for maintenance of cardiac function.

### Older adults

Aging results in profound cardiac changes, even in healthy individuals. With natural increases in blood pressure, greater stress is put on the left ventricle to pump blood, leading to myocardial hypertrophy and fibrosis (Lakatta and Levy, 2003b; Strait and Lakatta, 2012; Ugander et al., 2012; Liu et al., 2013). This inevitably leads to impairment of contractile and relaxation properties of the left ventricle (Strait and Lakatta, 2012), while age-related reductions to maximal HR limits maximum Q̇ (Lakatta and Levy, 2003b). Older adults also have higher risk of developing cardiac arrhythmias (Manolio et al., 1994), especially atrial fibrillation when atrial size is increased (Psaty et al., 1997). In a large study of enrolled adults up to 88 years of age, unfit individuals exhibited greater relative risk of cardiovascular-related deaths for both men (RR = 1.7) and women (RR = 2.42) (Blair et al., 1996). Furthermore, a recent meta-analysis confirmed the potent cardioprotective effects of aerobic training for maintenance of cardiac function in older men (>45 years) (Beaumont et al., 2019). Therefore, further investigation of exercise countermeasures during HDBR and extended periods of sedentary behaviour are essential.

## Blood volume

### Spaceflight

Early space research identified consistent losses to blood volume, with contraction of both plasma volume and red cell mass in-flight. During the Apollo program, astronauts landed with reductions in plasma volume of 4.4% and associated losses of 10% to red cell mass (Kimzey et al., 1975) This was subsequently followed by an immediate expansion of their intravascular volume by 4.8% after one day of recovery. Investigations of fluid balance during spaceflight are complicated by anecdotal reports of voluntary dehydration immediately prior to launch to prevent the need for urination, and by the possibility of space motion sickness with vomiting and reduced early fluid ingestion (Gharib and Hughson, 1992). Cephalad fluid shifts in microgravity and the subsequent negative fluid balance, fluid shifts between intravascular and interstitial spaces, and uncoupling of the kidney in space (Diedrich et al., 2007) (Table 1) all lead to a relative haemoconcentration during early spaceflight (Leach and Johnson, 1984). This uncoupling describes the lack of urinary fluid, sodium and urodilatin excretion on entry to microgravity despite elevated plasma atrial natriuretic peptide activity, as measured via urinary cyclic guanosine monophosphate excretion (Drummer et al., 2000). Although atrial natriuretic peptide activity is initially elevated, it subsequently falls whilst aldosterone and renin activity rise in-flight by day 100, a response to contraction of blood volume (Hughson et al., 2016). Despite evidence of reduced red cell production to correct an early haemoconcentration (Udden et al., 1995), later work suggests haemolysis of newly formed red blood cells (Alfrey et al., 1996) and/or persistent red cell haemolysis throughout flight can play an important role in haematological homeostasis (Trudel et al., 2022). Other than some evidence of pre-landing salt loading attenuating plasma volume losses (Leach et al., 1996), and the combination of exercise and salt loading preventing ambulatory orthostatic intolerance immediately post-flight (Fu et al., 2019), there is little evidence of the role of exercise alone in preventing blood losses during flight.

### Bedrest studies

There are differences in the mechanisms through which blood volume is regulated in space when compared to ground studies (Table 1). Importantly, the increases in left atrial pressure and subsequent Gauer-Henry reflex (Gauer et al., 1961) are short-lived or non-existent during spaceflight, whilst persisting in analogue studies with a concomitant and significant diuresis (Hughson and Bondar, 1999; Diedrich et al., 2007). However, the plasma volume losses are similar, so analogue studies do provide important evidence for the role of exercise during periods of inactivity and immobility, with translational results for both terrestrial and extraterrestrial environments. Nevertheless, analogue studies cannot reproduce the unloading of the thorax or reduction in CVP experienced in microgravity, as CVP initially rises during HDBR but then falls back to baseline levels within 20 h (Gaffney et al., 1985). Therefore, HDBR results must be interpreted whilst considering that the physiology leading to blood loss is different between HDBR and spaceflight.

In young adults, exercise modality and intensity appear to be an important determinants of blood volume outcomes during HDBR. Daily aerobic supine cycling at 75% maximal HR (3 sessions x 30-min/day) prevented plasma volume losses following HDBR (Shibasaki et al., 2003; Shibata et al., 2010). Additionally, one bout of maximal supine cycle exercise to exhaustion during HDBR resulted in a complete reversal of HDBR-induced plasma volume losses over the subsequent 24 h (Convertino et al., 1996). Countermeasures during the WISE-2005 study also proved beneficial (Guinet et al., 2009). Conversely, other exercise modalities appear to have less of a protective effect on blood volume. Isotonic (68% of maximal $\dot{V}$ ˙O_2_) and isometric exercise (21% maximal leg extension force) lasting half an hour each per day did not prevent plasma volume losses during two weeks of supine bedrest (Dolkas and Greenleaf, 1977). During HDBR, neither high-intensity jump training nor resistance vibration exercise showed any protective effects on blood volume (Kramer et al., 2017b; Guinet et al., 2020). The cross-over study design of Guinet et al. provides compelling evidence for the lack of benefit of lower-limb resistance exercise with concurrent vibration at 24Hz, as the exercise intervention failed to attenuate plasma losses of approximately 15% over 21 days of HDBR (Guinet et al., 2020). Given the noticeable differences in outcomes between studies, clarification of the type and intensity of exercises needed to maintain blood volume is an important step for adequate countermeasure activity in astronauts.

### Older adults

Blood volume has been reported to decline with age (Gibson and Evans, 1937; Davy and Seals, 1994; Jones et al., 1997), but others have not found such an association (Carrick-Ranson et al., 2013). Regardless of age, physical activity is a critical determinant of blood volume (Convertino, 2007), and by maintaining regular physical activity older adults have comparable blood volumes to younger active adults (Jones et al., 1997). Given the importance of blood volume in maintaining blood pressure during orthostatic challenge (Shibata et al., 2010) and carrying oxygen to active muscles (Jones et al., 1997; Lundgren et al., 2021), establishing countermeasures to preserve blood volume during periods of bedrest is critical to preserve physical function in older adults following immobility.

## Cerebrovascular function

### Spaceflight

Limited data exist on the adaptation of the cerebral vasculature and cerebral autoregulation (CA) to spaceflight. During a 16-day Neuro-lab mission, static CA remained intact with enhanced dynamic CA post-flight (Iwasaki et al., 2007). Middle cerebral artery velocity (MCAv) was unchanged during short-duration spaceflight (Arbeille et al., 2001; Iwasaki et al., 2007). Although evidence suggests that those who exhibit orthostatic intolerance (OI) following spaceflight could not regulate cerebral blood flow (determined through MCAv) in response to decreased mean arterial pressure at the level of the brain (Blaber et al., 2011). Long-duration spaceflight appears to impair dynamic CA, although static CA remained intact (Zuj et al., 2012). Cerebrovascular CO_2_ reactivity was also blunted, possibly as a consequence of the higher ambient CO_2_ aboard the ISS (Zuj et al., 2012). Resting supine mean arterial pressure at the level of the brain was maintained after long-duration flight (Zuj et al., 2012; Iwasaki et al., 2021); however, resting MCAv remained stable (Zuj et al., 2012) or increased (Iwasaki et al., 2021) compared to pre-flight values. During brief microgravity exposures, directly measured intracranial pressure decreased (Lawley et al., 2017) and estimated intracranial pressure remained unchanged after six months in space (Iwasaki et al., 2021). During resistive exercise in microgravity intracranial pressure did transiently increase, likely due to a Valsalva (Lawley et al., 2017). However, this repeated transient increase of intracranial pressure during resistive exercise appeared to have no impact on long-term estimated intracranial pressure measured after spaceflight (Iwasaki et al., 2021).

### Bedrest studies

The impact of exercise on cerebrovascular function during HDBR appears equivocal. Without exercise countermeasures, resting MCAv was increased (Kawai et al., 1993), maintained (Zhang et al., 1997; Arbeille et al., 2001; Pavy-Le Traon et al., 2002) or decreased (Frey et al., 1993; Arbeille et al., 1998) in HDBR studies ranging from 24 h to 42 days. Cerebrovascular resistance index (CVRi) was also increased during four-day HDBR (Arbeille et al., 1998), or maintained during seven (Pavy-Le Traon et al., 2002) and 42-day HDBR (Arbeille et al., 2001).

There does not appear to be any effect of resistance and/or aerobic exercise on end-tidal partial pressure of carbon dioxide (P_ET_CO_2_), MCAv, or CVRi during HDBR (Jeong et al., 2012, 2014; Kermorgant et al., 2019). The 60-day WISE-2005 study also identified no effect of exercise on cerebrovascular autoregulation, although all participants exhibited lower P_ET_CO_2_ with greater MCAv for any given P_ET_CO_2_ (Greaves et al., 2007). Aerobic cycling exercise had no impact (Jeong et al., 2012) or was detrimental to dynamic CA (Jeong et al., 2014), as was resistance vibration exercise (Kermorgant et al., 2019); other studies identified maintenance or even improvement of dynamic CA in sedentary control groups (Jeong et al., 2012; Kermorgant et al., 2019). Inter-individual variation appears to play a larger role in CA responses after HDBR rather than the exercise countermeasures themselves (Greaves et al., 2007). Overall, there is no clear effect of exercise on cerebrovascular function during HDBR.

### Older adults

Cerebral blood flow decreases with age (Graff et al., 2022), as MCAv is reduced and cerebrovascular resistance is increased (Zhu et al., 2011). Inefficient CA can also occur with aging, but CA is quite variable within the population of healthy older adults (Liu et al., 2016; de Jong et al., 2017). Furthermore, arterial stiffening impairs the ability for the brain to buffer changes in cerebral blood flow pulsatility, risking the development of cerebral small vessel disease (Tarumi and Zhang, 2018) and subsequent cognitive impairment, gait disturbances, and even stroke (Li et al., 2018). “Life-long” and short-term aerobic exercise helps preserve cerebrovascular function, including helping maintain cerebral blood flow, compared to sedentary individuals of the same age (Chapman et al., 2013; Tarumi et al., 2013; Thomas et al., 2013). Indeed after just 10 days of no exercise, older endurance athletes had a significant reduction in cerebral blood flow (Alfini et al., 2016). As aerobic exercise has a clear benefit to cerebrovascular function in older adults, further assessments of aerobic and resistance exercises, and their benefits on cerebrovascular function during HDBR are required.

## Vascular adaptions

### Spaceflight

Recently identified vascular adaptations to spaceflight occurred despite access to newer exercise equipment and current exercise countermeasure regimes aboard the ISS. Reduced pulse wave transit times to the finger (Baevsky et al., 2007; Hughson et al., 2016) and ankle (Hughson et al., 2016) after prolonged spaceflight indicates increased central and peripheral artery stiffness. Changes in common carotid artery (CCA) distensibility coefficient and *β*-stiffness index after six-month missions also suggest arterial stiffening (Hughson et al., 2016; Arbeille et al., 2017), with the magnitude equivalent to 10–20 years of normal aging on Earth (Kawasaki et al., 1987; Gepner et al., 2014). Similar changes in CCA distensibility and *β*-stiffness were also reported by Lee et al. (2020), although their chosen statistical methodologies did not achieve significance. Men had greater changes in pulse wave transit time and women had greater changes in *β*-stiffness (Hughson et al., 2016), but larger sample sizes are needed confirm these findings. Assessment of ultrasound backscatter energy from astronauts’ CCAs support a hypothesis of arterial wall remodeling with altered arterial wall content underlying observed changes in stiffness (Arbeille et al., 2021). Furthermore, insulin resistance and hyperglycemia were reported in astronauts after six months in space (Hughson et al., 2016), which could also contribute to changes in vascular properties (Webb et al., 2010).

Common carotid artery intima-media thickness (IMT), which is clinically used as a surrogate marker for atherosclerosis (Coll and Feinstein, 2008), increased by 12% over six months in space (Arbeille et al., 2016) and by ∼20% in the one-year NASA twin study (Garrett-Bakelman et al., 2019); however, results have not been consistent (Lee et al., 2020). Most of the increase in CCA IMT (10%) was reported to occur within the first 15 days of spaceflight (Arbeille et al., 2016), with similar changes (+15%) in the superficial femoral artery (SFA) (Arbeille et al., 2016). Interestingly, increases in CCA IMT were observed during the Mars-500 confinement study (Arbeille et al., 2014), where men lived isolated, albeit upright in normal gravity, suggesting that non-gravitational factors can influence IMT measurements (Arbeille et al., 2016). Brachial (Lee et al., 2020) and SFA (Arbeille et al., 2016) diameters change during long-duration spaceflight, with mixed outcomes for the CCA diameter, as both increases 5%–7% (Garrett-Bakelman et al., 2019; Lee et al., 2020), or no change have been reported (Arbeille et al., 2016). Furthermore, endothelium-dependent flow-mediated dilation (FMD) and endothelium-independent nitroglycerin vasodilation of the brachial artery were unchanged following flight (Lee et al., 2020). In summary, despite current exercise countermeasures, astronauts’ arteries become stiffer with increased IMT.

### Bedrest studies

Bedrest and physical inactivity are strong stimuli for vascular remodelling. It is well established that exercise and its associated haemodynamic responses enhance vascular function and structure, but distinct local vascular adaptations and benefits of exercise depend on the its modality, duration, and intensity (Green et al., 2017). To date, no prolonged bedrest study has evaluated the effects of exercise countermeasures on arterial stiffness. However, the effect of HDBR without exercise over 60 days, appears equivocal. Möstl et al. (2021) found no change in brachial-femoral pulse wave velocity, aortic distensibility, or corrected pulse arrival time for isovolumetric contraction time to different vascular beds (arm, finger, thigh, and toe). Furthermore, they stratified their sample into age tertiles, finding no evidence of increased risk for vascular changes in their older (37–55 years) compared to younger participants (24–36 years), despite higher brachial-femoral pulse wave velocity with greater age (Möstl et al., 2021). In contrast, Fayol et al. (2019) reported increased carotid-femoral pulse wave velocity during 60 days of HDBR, which remained elevated even after one year of recovery. HDBR of 35 days also did not result in changes to carotid-femoral pulse wave velocity (Palombo et al., 2015). Given the disparate responses between studies (Palombo et al., 2015; Fayol et al., 2019; Möstl et al., 2021), clarification of prolonged bedrest effects on central artery stiffness and the potential benefits of exercise are required. If eventually concluded that bed rest and spaceflight do not lead to similar changes in arterial stiffness, it may suggest that other factors, such as space radiation exposure, changes in blood glucose, or altered hormonal background (Hughson et al., 2018), are important factors in the vascular changes that occur during long-duration spaceflight.

Vascular remodelling occurs with the absence of exercise during supine or HDBR, leading to reduced diameters of arteries feeding locomotor muscle groups, including the common femoral (Bleeker et al., 2005; Palombo et al., 2015), SFA (Bleeker et al., 2005) and popliteal arteries (Dyson et al., 2007), but CCA diameter appears unaffected by bedrest (Bleeker et al., 2005; van Duijnhoven et al., 2010b; Palombo et al., 2015). Vascular diameter remodeling occurs rapidly and follows a non-linear time course, as during the first Berlin bedrest study, common femoral artery diameters reduced by ∼13% over the first 25 days of a 52-day supine bedrest campaign, and only fell by ∼4% more in the final 27 days (Bleeker et al., 2005). Lower-body resistive exercises were able to attenuate these decreases by ∼5% and ∼6% at days 25 and 52, respectively (Bleeker et al., 2005). Importantly, these exercises only provided locally protective effects, as brachial artery diameters fell in both control and exercisers alike (Bleeker et al., 2005). Subsequently, the second Berlin bedrest study revisited these findings, comparing lower-body resistive exercise with or without vibration. Exercises included squats (10 reps, 80% maximum force, ±20–24 Hz vibration), single-leg heel raises (to exhaustion, 1.3× body weight, ±26 Hz), double-leg heel raises (to exhaustion, 1.8× body weight, ±24 Hz) and back and heel raises (full extension for 60 s, 1.5× body weight, ±16 Hz) for three days every week. Again, resistance exercise attenuated reductions to SFA diameter, with greater attenuation in the vibration group (van Duijnhoven et al., 2010b). Results from the WISE-2005 study corroborated the protective effects of exercise on arterial remodelling (Dyson et al., 2007).

Following 35 days of HDBR without countermeasures, no change in CCA IMT was noted (Palombo et al., 2015). In the second Berlin bedrest study, thickening of the walls of the CCA (+17% IMT) and SFA (+13% IMT) were induced by 60 days of HDBR, whilst exercises with and without vibration helped prevent these increases (van Duijnhoven et al., 2010a). Given the magnitude of change in IMT in the control group over a short period of time (∼75 times greater than would be expected with 60 days aging), and the protective effects of exercise, IMT changes appear sensitive to activity level and do not indicate rapid development of atherosclerosis in these particular circumstances (van Duijnhoven et al., 2010a).

FMD responses from bedrest studies indicate paradoxical results (i.e., enhanced vasodilation suggesting improved endothelial function following prolonged inactivity), as responses in the leg were greater following 49-day HDBR (Platts et al., 2009). Exercise countermeasures during the WISE-2005 bedrest campaign (Dyson et al., 2007), and during the Berlin bedrest studies (Bleeker et al., 2005; van Duijnhoven et al., 2010b) were able to maintain or dampen increases in popliteal and SFA FMD responses after prolonged bedrest. The inverse relationship between resting artery diameter and proportional dilation (van Duijnhoven et al., 2010b) suggests that as arteries become smaller due to inactivity during bedrest, they dilate more. Therefore, in order to correctly interpret FMD findings during bedrest studies, an understanding of diameter-percent FMD relationships is critical (Thijssen et al., 2008).

The effect of bedrest on arterial stiffness remains unclear; however, regional artery dimensions are sensitive to inactivity. Exercise countermeasures have a critical role in maintaining vessel diameter but have focused primarily on lower-body exercise. Moving forward, multi-modal, whole-body exercise countermeasures that utilize both the upper and lower body should be explored to ensure that vascular protection is not localized to only certain branches of the vascular tree.

### Older adults

Older age is associated with increases to IMT (Karikkineth et al., 2020) and endothelial dysfunction (Seals et al., 2011). Additionally, arteries stiffen due to (amongst others) increased calcification, collagen production, and inflammation, resulting in increases to pulse-wave velocity and pulse pressure within vessels (Donato et al., 2018). This increases the risk of developing hypertension and cardiovascular disease (Mitchell et al., 2010). In sedentary but otherwise healthy older adults, both continuous aerobic training and resistance training multiple days a week for 8–12 weeks reduced arterial stiffness, but high-intensity interval training did not (Kim et al., 2017; Park et al., 2020; Akazawa et al., 2021). Notably, two weeks of bedrest in older adults impaired endothelial function (Goswami et al., 2015), but changes in other arterial properties of healthy older adults during bedrest, and the potential benefit of concomitant exercise remain unexplored.

## Autonomic changes

### Spaceflight

Autonomic control of the cardiovascular system is altered by spaceflight, and its function is critical for regulation of blood pressure, especially during postural changes. Following short-duration flights, vagal baroreflex responses were impaired (Fritsch et al., 1992; Fritsch-Yelle et al., 1994; Eckberg et al., 2010), and increases in peripheral resistance and norepinephrine were attenuated upon standing (Fritsch-Yelle et al., 1996). In-flight measures of vagal baroreflex sensitivity during the Neuro-lab mission were also reduced (Cox et al., 2002), and muscle sympathetic nerve activity (MSNA) and norepinephrine spillover were elevated in-flight and post-flight, with enhanced sympathetic responses observed to LBNP in space (Ertl et al., 2002). These findings suggest enhanced sympathetic activation in space and during the days following return to Earth. Importantly, the sympathetic reflex responses to orthostatic challenge following short-duration spaceflight (Levine et al., 2002; Beckers et al., 2009), and hand-grip and cold-pressor tests performed in-flight and post-flight (Fu et al., 2002) were appropriate in magnitude, which indicated that sympathetic mechanisms of cardiovascular control remained intact. Trends towards increased sympathetic activity during spaceflight were identified by the ratio of low-to-high frequency power from HR variability analysis, but are difficult to interpret due to postural and respiratory effects (Mandsager et al., 2015). Vagal baroreflex sensitivity was maintained during long-duration missions on the ISS in-flight, but reduced immediately upon landing (Hughson et al., 2012), albeit less than expected based on previous short-duration spaceflight findings (Fritsch et al., 1992; Fritsch-Yelle et al., 1994) and following 9 months on the Mir space station (Cooke et al., 2000). This decreased vagal activity abates quickly and may be appropriate for the stress of standing in astronauts immediately following landing (Beckers et al., 2009). Autonomic control of astronauts’ heart rate following both short- and long-duration spaceflight appeared to recover to pre-flight levels within 30 days of returning to Earth (Vandeput et al., 2013). Therefore, current in-flight exercise countermeasures help maintain cardiovascular control (Hughson et al., 2012).

### Bedrest studies

HDBR reduced parasympathetic nervous system activity (Hughson et al., 1994b; Crandall et al., 1994; Sigaudo et al., 1998), with mixed effects on the sympathetic nervous system. HR variability analysis suggests increased (Hughson et al., 1994b; Coupé et al., 2011) or unchanged sympathetic activity (Crandall et al., 1994; Sigaudo et al., 1998), while MSNA post-HDBR was increased (Kamiya et al., 2000; Barbic et al., 2019), unchanged (Pawelczyk et al., 2001) or decreased (Shoemaker et al., 1998). Interestingly, some individuals who presented with OI post-bedrest, had attenuated increases in MSNA (Kamiya et al., 2003), while others had normal MSNA responses to orthostatic stress, but reduced vasomotor response to sympathetic outflow (Arbeille et al., 2008). Similar to spaceflight, HDBR reduced vagal baroreflex response (Convertino et al., 1990; Hughson et al., 1994a; Sigaudo et al., 1998; Iwasaki et al., 2004). The reduction in plasma volume with bedrest has been implicated in the change of baroreflex sensitivity, as plasma volume restoration normalizes the spontaneous baroreflex function (Iwasaki et al., 2004). However, others reported that the change in baroreflex slope did not occur in parallel with changes to blood volume and persisted throughout the first week of recovery (Convertino et al., 1990), which suggests effects of bedrest on autonomic control independent of fluid status.

There are limited data available regarding the effectiveness of consistent exercise interventions alone to prevent the autonomic changes during and after HDBR. A single bout of dynamic and isometric high-intensity leg exercise, which leads to baroreceptor loading due to elevations in blood pressure, appeared to temporarily reverse reductions in vagal-mediated baroreflex function for ∼24 h following seven days of HDBR (Convertino et al., 1992). Other high-intensity resistance exercise interventions, such as reactive jump training (Maggioni et al., 2018) or resistance exercise combined with vibration (Coupé et al., 2011), appeared to help maintain resting sympathovagal balance following 60 days of HDBR. Resistance exercise combined with vibration slightly attenuated the reduction in baroreflex sensitivity following 60 days of HDBR (Coupé et al., 2011). Alternatively, the combination of aerobic exercise and artificial gravity was very effective at attenuating changes parasympathetic activity and baroreflex function post-bedrest (Iwasaki et al., 2005); however, delaying the start of artificial gravity and high-intensity exercise countermeasures by seven days, or possibly insufficient time dedicated to the countermeasure, reduced its effectiveness (Hughson et al., 1994a).

Overall, changes in autonomic cardiovascular control during and following HDBR involves many complex interactions between different parts of the cardiovascular system, leading to variable responses. Exercise alone transiently improves indices of autonomic function and can provide some benefit for maintaining baroreflex sensitivity and sympathovagal balance.

### Older adults

Autonomic function tends to decline with age, as sympathetic activity increases (Seals and Esler, 2000), and vagal baroreflex sensitivity is reduced (Ebert et al., 1992). Research indicates that aerobic exercise training improves cardiac autonomic control in older adults, determined by heart rate variability (Grässler et al., 2021), as well as baroreflex sensitivity (Madden et al., 2010). High-intensity interval training has been reported to confer similar autonomic benefits in older adults (Pichot et al., 2005), although others have not found an effect (Cassidy et al., 2019). Accordingly, it is unknown if exercise countermeasures are also effective at maintaining autonomic function in older adults during periods of immobility.

## Glucose handling

### Spaceflight

Early spaceflight indicated potential alterations to glucose handling over the course of a 150-days Salyut 7-Soyuz T9 sojourn, with delayed time to peak blood glucose during oral glucose tolerance test (OGTT) by flight day 60 through to post-flight day 25 in one cosmonaut (Alexandrov et al., 1985). OGTT responses in one astronaut whilst aboard the Mir space station also indicated greater plasma insulin and glucose concentrations compared to pre-flight responses (Macho et al., 2003). Later results from shuttle flights supported these findings, with measurements of c-peptide suggestive of increased insulin secretion to overcome in-flight insulin resistance (Stein et al., 1994), although these data were later combined with further flight data that questioned this initial finding (Stein et al., 1999b). The Spacelab D2 mission (1993) included OGTT assessment of four astronauts, finding higher mean glucose, insulin, and c-peptide concentrations in-flight, despite them remaining within normal ranges (Maaß et al., 1997). It was not until recently that statistically significant increases in insulin resistance index and close-to-significant increases in glycated albumin were confirmed in both male and female astronauts aboard the ISS (Hughson et al., 2016). Importantly, these data identified altered glucose handling in astronauts performing the most up-to-date exercise countermeasure activities. However, consideration of astronauts’ absolute exercise duration must be made (Fraser et al., 2012; Hughson et al., 2016). Thus, the current duration, intensity, and/or modality of exercise during spaceflight appears to be insufficient to prevent altered glucose handing given the limited data we have from space.

### Bedrest studies

HDBR is known to cause impaired insulin sensitivity as documented in several different studies (Blanc et al., 2000; Heer et al., 2014; Montero et al., 2018; Rudwill et al., 2018). However, only a small number of investigations studying the beneficial effects of exercise on insulin resistance exist. Isotonic and isometric exercises reduced insulin and glucose responses to OGTT compared to control subjects in a three-arm cross-over supine bedrest and were inversely proportional to energy expenditure during each campaign (Dolkas and Greenleaf, 1977). During long-duration HDBR of 60 and 70 days, both resistive vibration exercise (Yang et al., 2014) and a combination of resistive and aerobic exercise (Downs et al., 2020) attenuated the detrimental effects on insulin tolerance. Potential mechanisms include transmembrane GLUT-4 content in skeletal muscle, as maximal isometric leg-press exercises increased concentrations in VL during 19-day HDBR, whilst control participants experienced relative decreases (Tabata et al., 1999). However, ongoing research into the potentially protective role of exercise during periods of HDBR is needed.

### Older adults

Incidence of insulin resistance increases with age, with reasons including reduced physical activity (Bauman et al., 2016; McGlory et al., 2018), and intrinsic changes related to skeletal muscle aging (Shou et al., 2020). However, consistent physical activity with little sedentary time appears to improve glucose metabolism in older adults (Länsitie et al., 2021). Both endurance and resistance training improve glucose metabolism in older adults, especially in those who were previously sedentary (Consitt et al., 2019). High-intensity interval training can also enhance glycemic control (Cassidy et al., 2016). Even as little as 2000 steps/day in older adults undergoing seven days of bedrest preserved glucose metabolism in older adults (Arentson-Lantz et al., 2019). Considering this, glucose handling assessment in older populations during HDBR with exercise countermeasure interventions may provide clinically important information that could inform medical practice.

## Orthostatic intolerance

Orthostatic intolerance occurs when delivery of oxygen to the brain in upright posture is insufficient resulting in symptomatology, including light-headedness, dizziness, and even syncope (Van Lieshout et al., 2003). Spaceflight and bedrest both induce cardiovascular changes that hinder the supply of oxygen to the brain during orthostatic stress. Mechanisms contributing to OI after spaceflight or bedrest are multifaceted, including failure to maintain arterial blood pressure due to changes in cardiac function coupled with reduced blood volume and venous return (Levine et al., 1997; Perhonen et al., 2001b; Shibata et al., 2010) or inadequate arterial vasoconstriction (Buckey et al., 1996b; Waters et al., 2002), as well as impaired CA (Zhang et al., 1997). However, OI has been identified in cases with maintained arterial blood pressure but paradoxical cerebral vasoconstriction (Grubb, 2005; Novak, 2016).

### Spaceflight

OI was first noted during Mercury missions, with symptom severity dependent on mission duration (Link, 1965). OI continued to be reported following longer 14-day Gemini flights (Berry et al., 1966), where noticeable inter-individual variability was reported (Berry and Catterson, 1967). Even with the introduction of routine onboard exercise countermeasures, OI persists, with increasing prevalence at landing as mission length increases (Meck et al., 2001). Despite access to the iRED and TVIS devices, ISS astronauts who flew for ∼177 days had far greater rates of OI compared to shuttle astronauts flying for ∼17 days (Lee et al., 2015). Additionally, recent studies of eight long-duration astronauts identified two that experienced orthostatic hypotension during a three-minute stand test (Wood et al., 2019). As formal orthostatic testing equates to motionless standing without muscle contraction, Fu et al. (2019) assessed OI during activities of daily living in 12 astronauts following approximately six months in space (between 2009–2013) finding no evidence of OI or orthostatic hypotension. Therefore, standing still following landing should be discouraged in returning astronauts, and activity encouraged as a countermeasure. Another possible way to reduce risk of OI is *via* fluid loading immediately prior to landing (Bungo et al., 1985). During shuttle missions, approximately one-half of the astronauts used such a protocol, but risk of OI was unaffected (Buckey et al., 1996b). Today, flight surgeons recommend fluid loading prior to landing, but there are no systematic data to evaluate the efficacy of this countermeasure. With regards to identifying “at-risk” individuals, tolerance to upright posture following landing has great inter-individual variability (Buckey et al., 1996b; Levine et al., 2002; Meck et al., 2004; Blaber et al., 2011). Women are more likely to experience OI after spaceflight, possibly related to differences in CA, or lower peripheral vascular resistance and hypo-adrenergic responses (Fritsch-Yelle et al., 1996; Waters et al., 2002; Blaber et al., 2011), but prediction otherwise remains difficult.

### Bedrest studies

Without countermeasures, prevalence of OI during orthostatic testing increased after as little as four-day HDBR (Arbeille et al., 1998). Similar results were observed following seven (Custaud et al., 2002), 14 (Zhang et al., 1997; Grenon et al., 2004), 21 (Barbic et al., 2019), 42 (Pavy-Le Traon et al., 1998), and 60 days (Liu et al., 2015). Unlike spaceflight, rates of OI were similar between men and women (Custaud et al., 2002), although only a few studies have included both sexes in the experimental design (Watenpaugh et al., 2007).

Exercise alone during HDBR does not appear to provide complete protection from OI. Varied aerobic rowing and resistance exercise 6-days/week during 35-day HDBR (Hastings et al., 2012) or 90-minutes of daily aerobic cycling at 75% maximum HR during 18-day HDBR (Shibata et al., 2010; Jeong et al., 2012) both failed to preserve orthostatic tolerance. However, when combined with plasma volume restoration, orthostatic tolerance was preserved (Shibata et al., 2010; Hastings et al., 2012; Jeong et al., 2012). Surprisingly, the aforementioned aerobic cycling actually exaggerated OI following HDBR compared to controls (Shibata et al., 2010; Jeong et al., 2012). Resistance vibration exercise was not found to have any impact on reducing OI compared to a sedentary control group after 21 days (Guinet et al., 2020) and 90 days of HDBR (Belin de Chantemèle et al., 2004). The introduction of a gravitational stimulus using supine treadmill exercise inside LBNP attenuated the reduction (-13%) in orthostatic tolerance time compared to controls (−34%) following 30 days of bedrest (Watenpaugh et al., 2007). The WISE-2005 study utilized similar exercise countermeasures but appeared less effective, as orthostatic tolerance time was reduced by 35% in the countermeasure group compared to 50% in controls, although a lack of exercise in the final 62 h may have confounded these results (Guinet et al., 2009). Overall, the ability of exercise to attenuate OI after HDBR is complex and dependent on synergistic effects with other simultaneous countermeasures. Furthermore, individual physical and physiological factors are likely playing a role in OI susceptibility (Pavy-Le Traon et al., 1999), making it difficult to draw concrete conclusions.

### Older adults

In adults over the age of 60, one in five experience OI, which increases to approximately one in four if residing in long-term care (Saedon et al., 2020). Presence of OI is associated with increased risk of falls (Finucane et al., 2017), cardiovascular and cerebrovascular morbidities (Ricci et al., 2015), and frailty (O’Connell et al., 2015). Mild to moderate exercise is often encouraged as part of an OI management plan (Figueroa et al., 2010), as OI is exacerbated by inactivity. With the low cost and ease in prescribing potentially effective exercise countermeasures, assessment of the efficacy of exercise in older adults is important, especially during periods of protracted immobility.

## Aerobic fitness

The aerobic response to exercise provides important information about the integrative physiological effects of microgravity and bedrest on multiple physiological systems. The marked cardiovascular and musculoskeletal changes during spaceflight and inactivity alter the oxygen transport cascade and oxygen utilization. Reduced blood volume limits potential increases to Q̇ due to impaired cardiac filling, reduced SV and elevated resting HR (Convertino, 1997), while decreased conduit artery diameters further limit oxygen delivery to exercising muscles (Dinenno et al., 2001; Miyachi et al., 2001). Loss of muscle mass and myofiber shifts to less oxidative types impair aerobic metabolism (Trappe et al., 2009). Even orthostasis (upright vs. supine) can impair the supply of oxygen to working muscles post-bedrest (Convertino et al., 1982; Hung et al., 1983). A simple integrative outcome assessing these whole-body physiological consequences and the potential benefit of exercise is maximal rate of oxygen uptake ( $\dot{V}$ O_2_max).

### Spaceflight

It was identified early in human spaceflight that exercise countermeasures were needed to attenuate deconditioning and preserve work ability [see review by Moore et al. (Moore et al., 2010)]. Limited $\dot{V}$ O_2_max data were collected during early missions, but submaximal $\dot{V}$ O_2_ and HR data were suitable alternatives. Following short-duration Gemini and Apollo missions, reductions in aerobic fitness were inferred from large reductions in $\dot{V}$ O_2_ (19%–26%) and work rate for a given HR post-flight (Dietlein and Rapp, 1966; Berry and Catterson, 1967; Rummel et al., 1973). This prompted the implementation of more extensive exercise countermeasures (see Figure 2) with allocated daily exercise of 30–90 min during the Skylab missions (Johnston, 1977). Increasing exercise intensity and duration better maintained astronauts’ fitness in-flight (Michel et al., 1975, 1977; Rummel et al., 1976) and post-flight (Greenisen et al., 1999), while limited exercise during shuttle missions due to time constraints resulted in decreases to post-flight fitness (Levine et al., 1996).

During six-month missions to the ISS, average $\dot{V}$ O_2_max (−17%) and peak work rate (−24%) fell early in-flight, gradually recovering but never reaching pre-flight levels (Moore et al., 2014). Immediately post-flight, $\dot{V}$ O_2_max was reduced (−15%), returning to pre-flight levels within 30 days of landing (Moore et al., 2014; Lee et al., 2020). However, there was large variation in the effectiveness of the exercise countermeasures between astronauts; despite reductions to average $\dot{V}$ O_2_max, half of those studied achieved in-flight values at or above pre-flight levels, with a few subsequently experiencing only minimal reductions at landing (Moore et al., 2014). Fitter individuals had the greatest reduction in V̇O_2_max, while those performing high-intensity exercise training (>80% maximum HR on CEVIS and treadmill), or those with relatively lower pre-flight fitness maintained fitness during flight (Moore et al., 2014). High intensity-low volume training appears to confer similar benefits for aerobic fitness (English et al., 2020). Therefore, spaceflight-induced reductions in aerobic fitness can be eliminated if exercise countermeasures are properly prescribed.

### Bedrest studies

Aerobic fitness appears to decay during bedrest relatively linearly, as revealed in a recent meta-analysis [0.43% (−0.22 ml/kg/min) per day] (Ried-Larsen et al., 2017), although the rate of decay might be faster in the first 10 days (Capelli et al., 2006; Ade et al., 2015). As with spaceflight, individuals with higher pre-bedrest $\dot{V}$ O_2_max lose more when compared to lower fitness individuals (Ried-Larsen et al., 2017). Following shorter duration bedrest studies (up-to two weeks), individuals typically fully recover fitness within one week (Trappe et al., 2006), with slower recovery following longer duration bedrest (Sundblad et al., 2000).

Various exercise countermeasures implemented during bedrest studies attempted to preserve aerobic fitness. Low-intensity aerobic exercise (40% $\dot{V}$ O_2_max) up to one hour daily was insufficient to prevent loss of aerobic fitness (Suzuki et al., 1994), whilst an hour of daily moderate-intensity (68% of $\dot{V}$ ˙O_2_max) exercise partially attenuated reductions in $\dot{V}$ O_2_max (Stremel et al., 1976). Therefore, higher intensity or larger training volumes are required to maintain aerobic fitness during bedrest. Ninety minutes of daily cycling exercise (75% maximum heart rate) prevented losses to $\dot{V}$ O_2_max after 18 days of bedrest (Shibasaki et al., 2003), while two 30-min high-intensity interval training sessions performed five days per week maintained $\dot{V}$ O_2_max over a 30-day bedrest campaign (Greenleaf et al., 1989). Daily aerobic exercise coupled with volume loading at the end of bedrest to restore plasma volume maintained $\dot{V}$ O_2_max (Shibata et al., 2010). Resistance exercise attenuated reductions in $\dot{V}$ O_2_max (Stremel et al., 1976), and a combination of lower-body resistance exercise and whole-body vibration prevented significant reductions in $\dot{V}$ O_2_max (Guinet et al., 2020). One hour or less of total daily exercise, consisting of high-intensity interval training in combination with continuous aerobic and resistance training, maintained or even improved $\dot{V}$ O_2_max following 14 days of bed rest (Ploutz-Snyder et al., 2014). Another effective high-intensity, low-volume exercise type is sledge jumping, as 48 exercise sessions (3 min of exercise per session) spread over 60 days of HDBR prevented reductions in aerobic fitness (Kramer et al., 2017a). More recently, hybrid countermeasures combining both exercise and artificial gravity garnered more research focus, as exercise and artificial gravity appear to work synergistically to maintain fitness and attenuate cardiovascular changes. The use of centrifugation (Katayama et al., 2004) or LBNP (Watenpaugh et al., 2000; Lee et al., 2007) with simultaneous high-intensity interval exercise has been very effective at maintaining aerobic fitness post-bedrest. Moderate-intensity cycling during artificial gravity also maintained $\dot{V}$ O_2_max during four-day HDBR (Li et al., 2017). The addition of resistance training to aerobic training with LBNP also maintained aerobic fitness post-HDBR (Schneider et al., 2009). However, artificial gravity in the absence of exercise was not able to attenuate the bedrest associated reductions in $\dot{V}$ O_2_max (Kramer et al., 2021).

Given the relatively limited access to exercise equipment capable of simultaneous application of artificial gravity, both in space and on Earth, high-intensity interval exercise in combination with resistance training currently appears to be the most feasible countermeasure to attenuate loss of aerobic fitness.

### Older adults

Older adults generally have lower fitness than younger adults (Betik and Hepple, 2008), but still experience larger reductions in aerobic fitness (12%–15%) after relatively short-duration (10–14 days) bedrest (Kortebein et al., 2008; Pišot et al., 2016). Physical activity levels tend to decline in older age (Hansen et al., 2019), but even those who remain as physically active as their younger counterparts exhibit lower average aerobic fitness (Aspenes et al., 2011). Immobility or bedrest can exacerbate losses in $\dot{V}$ O_2_max, leading to a decline in physical function (Coker et al., 2015). Aerobic and resistance exercises are strongly recommended for older adults in order to maximize aerobic fitness and reduce frailty (Izquierdo et al., 2021). Therefore, it is important to assess exercise regimes consisting of aerobic and resistance components and their ability to attenuate the loss in aerobic fitness in older adults during bedrest.

## Considerations for the upcoming bedrest study in older adults

### Older adult bedrest studies

To date, very few bedrest investigations in older adults have been undertaken, with most focussing on musculoskeletal consequences of bedrest without the inclusion of exercise countermeasures (Di Girolamo et al., 2021). Furthermore, the supine bed rest modality utilized in these studies does not invoke the same cardiovascular responses as those induced by 6° HDBR; indeed, HDBR in older adults is as-of-yet under-investigated.

Following supine bedrest, older adults show greater magnitudes of lower-limb muscle mass loss compared to younger adults, with reductions in muscle power that correlated with bedrest duration (Di Girolamo et al., 2021). After only 10-day bedrest, older adults already showed functional losses that include reduced stair-climbing and floor transfer times (Ferrando et al., 2010). Older adults also experienced greater proportional reductions in $\dot{V}$ O_2_max compared to younger adults for the same duration of bedrest (Pišot et al., 2016). Bedrest-induced cardiovascular changes in older adults are underexplored, although orthostatic intolerance (Pišot et al., 2016) and reduced endothelial function (Goswami et al., 2015) were reported following two-week supine bedrest. Magnitudes of other cardiovascular changes are relatively unknown, and must be evaluated given the age-related increases to rate of arterial stiffening with aging (Gepner et al., 2014) and greater cardiovascular disease risk (Lakatta and Levy, 2003a; 2003b). The detrimental and understudied physiological consequences of prolonged bedrest in older adults underscores the need for further identification and evaluation of appropriate exercise countermeasures to mitigate or prevent potentially pathological changes in this population.

### Goals for the bedrest study in older adults study

As evidenced by the preceding review, exercise can help mitigate or completely prevent many musculoskeletal and cardiovascular consequences of short- and long-term spaceflight and bedrest. However, the majority of bedrest and HDBR studies have been conducted in a young population (Ried-Larsen et al., 2017) and older adult bedrest studies have not included exercise countermeasures without participants leaving their beds (Di Girolamo et al., 2021). Furthermore, a suite of countermeasures must be assessed, as any one modality appears insufficient to alleviate detrimental consequences in all physiological systems. It is important to explore exercise countermeasures in older adult bedrest studies for their clinical relevance as well as observations of astronauts older than 50 years of age participating in critical space missions (Kovacs and Shadden, 2017) and future space tourists. To date, there are not systematic data comparing younger and older astronauts. Debatably, the older population is most affected by long-term sedentary bedrest, such as during hospital stays, which can reduce physical fitness leading to a greater risk of adverse outcomes (Kortebein et al., 2008; Venturelli et al., 2012). Therefore, an important research question is apparent: does exercise reduce the negative musculoskeletal and cardiovascular impacts of HDBR in older adults? In a clinical trial funded by the Canadian Institutes for Health Research, the Canadian Frailty Network, and Canadian Space Agency (NCT04964999), we propose assessing the effectiveness of a combination of high-intensity interval training (HIIT), continuous aerobic exercise, and upper- and lower-body resistance exercise to reduce musculoskeletal and cardiovascular deconditioning over 14 days of HDBR in older adults aged 55–65 years. The large multidisciplinary research team participating in the BROA study comprises experts in musculoskeletal and cardiovascular physiology, in addition to nutritional, cognitive, and medical specialists, and includes a wide range of hypotheses and investigative outcomes. Over the course of the following months and years, publications of exercise intervention efficacy in multiple physiological systems will further our understanding of potential countermeasure and rehabilitation practices aiding both terrestrial and extra-terrestrial individuals.

### Exercise countermeasure protocol rationale

Twenty to 24 participants will be randomly assigned to one of two groups, sedentary control, or exercise. The sedentary control group will participate in daily physiotherapy stretching sessions, whereas the exercise group will participate in three daily training sessions all while maintaining six-degree head-down tilt (Table 2). The countermeasure program was designed to address several key physiological systems affected by bedrest or spaceflight. Three exercise sessions per day totalling one hour with four hours between sessions were included, as breaking up sedentary time is beneficial to cardiometabolic health (Healy et al., 2008; Bergouignan et al., 2016). HIIT was added to the protocol for several reasons. High-intensity training has beneficial effects on maintaining or improving aerobic fitness. HIIT was demonstrated by Cassidy et al. (Cassidy et al., 2016) to induce robust metabolic and cardiovascular benefits in clinical populations with type 2 diabetes. As well, inclusion of high-intensity exercise might achieve the benefits on baroreflex response reported by Convertino (Convertino et al., 1992), although HIIT per se has had mixed effects showing benefit (Pichot et al., 2005) and no effect (Cassidy et al., 2019). Overall, the cycling exercises and upper- and lower-body resistance protocols were chosen to resemble high-intensity, low-volume programs which have found some success in maintaining musculoskeletal and cardiovascular outcomes during spaceflight and HDBR in a younger population (Ploutz-Snyder et al., 2014, 2018; English et al., 2020). Although not discussed within the scope of this review, nutritional intake will be controlled according to the Guidelines for Standardization of Bedrest Studies in the Spaceflight Context in order to help maintain body weight (Angerer et al., 2014).

**TABLE 2:** Proposed exercise countermeasure protocol for the bedrest study in older adults.

Week #1	Day 1	Day 2	Day 3	Day4	Day 5	Day 6	Day 7
Session 1	Resistance, Upper	Cont. Aerobic (30)	Progressive Aerobic	Cont. Aerobic (30)	HIIT	Cont. Aerobic (30)	HIIT
Session 2	Progressive Aerobic	Resistance, Lower	Cont. Aerobic (15)	Resistance, Upper	Progressive Aerobic	Resistance, Upper	Cont. Aerobic (15)
Session 3	HIIT	Progressive Aerobic	HIIT	Progressive Aerobic	Resistance, Lower	Progressive Aerobic	Progressive Aerobic

**Week #2**	**Day 8**	**Day 9**	**Day 10**	**Day 11**	**Day 12**	**Day 13**	**Day 14**
Session 1	Cont. Aerobic (15)	HIIT	Cont. Aerobic (30)	HIIT	Cont. Aerobic (30)	HIIT	Cont. Aerobic (15)
Session 2	Resistance, Upper	Cont. Aerobic (15)	Resistance, Lower	Cont. Aerobic (15)	Resistance, Lower	Progressive Aerobic	Cont. Aerobic (30)
Session 3	Progressive Aerobic	Progressive Aerobic	Progressive Aerobic	Progressive Aerobic	Progressive Aerobic	Resistance, Upper	Progressive Aerobic

Exercise type	Exercise time (min)	Equipment	Exercise description
HIIT	32	Cycle ergometer	5 min warm-up (40% HRR), 11 intervals (30s on @ 80%–90% HRR, 1.5 min relaxed cycling), 5 min cool-down
Cont. Aerobic (15)	15	Cycle ergometer	3 min warm-up (40% HRR), 9 min steady-state (60%–70% HRR), 3min cool-down (40% HRR)
Cont. Aerobic (30)	30	Cycle ergometer	5 min warm-up (40%–50% HRR), 20 min steady-state (60%–70% HRR), 5 min cooldown (40%–50% HRR)
Progressive Aerobic	15	Cycle ergometer	3 min stages at: 30%, 40%, 50%, 60%, 40% HRR
Resistance, Lower	25	Cables, resistance bands, body weight	3 sets (1 warm-up) of 10–12 repetitions
			Exercises: Hip raise, leg press, ankle pump, leg curls
Resistance, Upper	25	Cables, resistance bands, body weight	3 sets (1 warm-up) of 10–12 repetitions
			Exercises: External shoulder rotation, chest fly, lateral pull-down, dead bug

HRR, heart rate reserve.

## Conclusion

Spaceflight and HDBR without the use of exercise countermeasures cause loss of muscle mass and bone density, and marked deconditioning of the integrative function of the cardiovascular system, resulting in orthostatic intolerance and reduced aerobic fitness. Spaceflight studies conducted on middle-aged adults and HDBR studies focusing on young healthy adults demonstrated that a combination of aerobic and resistance exercise is best at maintaining or even improving musculoskeletal and cardiovascular systems variables. Exercise countermeasures during spaceflight or HDBR have beneficial effects on muscle mass and strength, muscle protein synthesis, cardiac morphology, and function, and vascular properties, leading to better aerobic fitness and orthostatic tolerance. However, the impact of exercise during spaceflight or HDBR on blood volume, cerebrovascular function, and autonomic function appears equivocal and should be the focus of future work. Furthermore, as we explain in this review, any one modality of exercise fails to attenuate all negative effects of spaceflight or HDBR. Indeed, despite the reported beneficial effects of specific exercise on isolated muscle groups or individual physiological systems, whole-body benefits appear limited for many of these countermeasure modalities. The need to formulate comprehensive and efficacious countermeasure regimes is paramount to ensure a holistic approach for both astronauts and immobile individuals throughout society. Finally, the current literature fails to address the effectiveness of exercise countermeasures during HDBR in older adults, even though they are more likely to be bed ridden, for example, during hospitalization. The bedrest-related decline in multiple physiological systems is exacerbated by age, necessitating assessment of exercise countermeasure interventions aimed at mitigating these changes. Therefore, we propose the BROA study, in which healthy older men and women, ages 55–65 years, will complete a 14-day HDBR campaign, with half the participants performing daily exercise during HDBR consisting of HIIT, aerobic, and resistance training. We hope the results from the BROA study will inform healthcare decisions that are especially important with transitions in care in older adults (Canadian Institutes of Health Research, 2021), and improve physiological outcomes on Earth and in space.
